# Familial autoimmunity in the childhood arthritis and rheumatology research alliance registry

**DOI:** 10.1186/s12969-016-0075-7

**Published:** 2016-03-10

**Authors:** Sampath Prahalad, Courtney E. McCracken, Lori A. Ponder, Sheila T. Angeles-Han, Kelly A. Rouster Stevens, Larry B. Vogler, Carl D. Langefeld, Susan D. Thompson, for The CARRA Registry Investigators

**Affiliations:** Department of Pediatrics, Emory University School of Medicine, 1760 Haygood Dr NE, Atlanta, GA 30322 USA; Children’s Healthcare of Atlanta, Atlanta, GA USA; Center for Public Health Genomics and Department of Biostatistical Sciences, Wake Forest School of Medicine, Winston-Salem, NC 27157 USA; Center for Autoimmune Genomics and Etiology, Cincinnati Children’s Hospital Medical Center, Cincinnati, OH USA

## Abstract

**Background:**

Clinically distinct autoimmune phenotypes share genetic susceptibility factors. We investigated the prevalence of familial autoimmunity among subjects with juvenile idiopathic arthritis (JIA), childhood systemic lupus erythematosus (cSLE) and juvenile dermatomyositis (JDM) in the CARRA Registry, the largest multicenter observational Registry for pediatric rheumatic disease.

**Methods:**

Children with JIA, cSLE and JDM enrolled in the CARRA Registry between May 2010 and May 2012 were investigated for differences in proportion of subjects who had first-degree relatives (FDR) with autoimmunity. If a significant difference was detected, pairwise comparisons, adjusted for multiple comparisons, were made.

**Results:**

There were 4677 JIA, 639 cSLE and 440 JDM subjects. The proportion of subjects having FDR with any autoimmune disease in the JDM group (20.5 %) was less compared to subjects with JIA (31.8 %, *p* < 0.001) or SLE (31.9 %; *p* < 0.001). Significantly greater proportion of JIA cases had FDR with inflammatory arthritis (13 %) compared to cSLE (9.2 %, *p* = 0.007) or JDM (4.3 %, *p* <0.001). Significantly greater proportion of cSLE cases had FDR with SLE (11.1 % vs. 1.7 % for JIA and 1.1 % for JDM *p* < 0.001) or type-I diabetes (7.4 % for cSLE vs. 3.1 % for JIA and 3.0 % for JDM *p* < 0.001).

**Conclusion:**

Higher proportions of subjects with JIA and cSLE have FDR with autoimmunity compared to those of JDM. Relatives of cSLE cases had an increased prevalence of SLE, and relatives of JIA cases were enriched for inflammatory arthropathies demonstrating distinct patterns of familial autoimmunity among these phenotypes.

## Background

Autoimmune disorders are relatively common, estimated to affect 5 to 10 % of the population. Juvenile idiopathic arthritis (JIA), childhood onset systemic lupus erythematosus (cSLE) and juvenile dermatomyositis (JDM) are examples of autoimmune disorders affecting the pediatric population. Although autoimmune disorders are clinically distinct phenotypes, there is substantial evidence to suggest that they may share common genetic susceptibility factors. Variants in *PTPN22* and *STAT4* genes exemplify genetic variants associated with multiple autoimmune phenotypes [1–4].

There is also clinical evidence for the clustering of autoimmunity in individuals and families [5, 6]. For instance, children with JIA have increased prevalence of autoimmune thyroiditis and type 1 diabetes [7, 8]. Relatives of children with JIA,, cSLE and JDM have a higher prevalence of autoimmunity [9–11]. In some instances, two autoimmune phenotypes show inverse relationships. While RA, autoimmune thyroiditis and type 1 diabetes were comorbid more often than expected, there was reduced comorbidity of multiple sclerosis and RA [6]. Our objective was to investigate the prevalence of familial autoimmunity among subjects with JIA, cSLE and JDM in the CARRA Registry, the largest multicenter observational Registry for pediatric rheumatic disease. We sought to explore if there were distinct patterns of familial clustering of autoimmune diseases among these phenotypes

## Subjects and methods

Children with JIA, cSLE and JDM from pediatric rheumatology clinics in the US were enrolled in the CARRA Registry between May 2010 and May 2012. Demographic and disease-related data were collected from time of diagnosis to enrollment visit as previously described [12–14]. Demographic variables including gender, race and ethnicity, as well as quality of life measures were collected by self-report by patients and/or parents via a questionnaire. Family history information included the presence of an autoimmune disease in any first degree relative of the proband. Specifically the following disorders was queried: SLE, psoriasis, Crohn’s disease or ulcerative colitis, ankylosing spondylitis/spondyloarthropathy, acute anterior uveitis, JIA, RA, multiple sclerosis, autoimmune thyroiditis, celiac disease, and type 1 diabetes. In addition, an “other autoimmune disorders” category was included. ANA status, age at onset of disease, age at diagnosis and age at enrollment were available by chart review. Institutional Review Board approval was given for this study by Emory University. CARRA sites also had local IRB approval to provide data to the CARRA Registry.

### Statistical methods

All statistical analyses were conducting using SAS 9.3 (Cary, NC). Statistical significance was assessed at 0.05 unless otherwise noted. Demographic and disease characteristics are summarized using means and standard deviations or counts and frequencies for each autoimmune disorder (subgroup), as appropriate. Demographic characteristics were compared among the three groups using one-way analysis of variance (ANOVA) models for continuous data and Chi-square tests for categorical data. For each autoimmune disorder (JIA, cSLE, and JDM), we calculated the proportion of children that had a first-degree relative with any type of autoimmunity. To determine if subjects with JIA, cSLE, or JDM had a different proportion of first-degree relatives with autoimmunity, we performed Chi-square tests or Fisher Exact tests if the frequencies were small (*n* < 5). When a significant difference was detected, post-hoc pairwise comparisons, adjusted for multiple comparisons, were made among the three groups to determine which groups significantly differed using a significance level of 0.05/3 = 0.017. Additionally, Chi-square tests were used to compare the proportion of children within JIA categories [15] (systemic, rheumatoid factor (RF)-negative polyarticular JIA, RF-positive polyarticular JIA, persistent oligoarticular JIA, extended oligoarticular JIA, psoriatic JIA, ERA, and undifferentiated JIA) that had a first-degree relative with autoimmunity. While we investigated if overall differences in the prevalence of familial autoimmunity existed between the different categories of JIA, we were not powered to perform pairwise comparisons between sets of JIA categories. Finally, the total number of familial autoimmune diseases was tabulated for each patient. The Kruskal-Wallis one-way analysis of variance was used to determine if number of familial autoimmune diseases significantly differed by disease group. Additionally, the number of familial autoimmune diseases was categorized into the following categories: 0, 1–2, 3 or more and compared among the three disease groups.

## Results

### Patient characteristics

The sample consisted of 5756 patients with JIA (*n* = 4677), cSLE (*n* = 639) or JDM (*n* = 440) from the CARRA registry. The majority of the patients were classified as having JIA (81.3 %), being female (73.3 %) and Caucasian (85.3 %). The average age of patients in the dataset at enrollment was 11.9 years (±4.8) and the average age at onset of symptoms was 7.1 years (±4.7). Table 1 provides a summary of patient demographics for the entire sample and broken down by disease group (JIA, cSLE and JDM). Patients with a diagnosis of JDM or JIA were younger than patients with a diagnosis of cSLE (JDM: 11.9 ± 4.4 years; JIA: 11.0 ± 4.4; cSLE: 16.0 ± 3.2 years; *p* < 0.001) and had a younger-age at onset of symptoms (*p* < 0.001). Additionally, cSLE patients were more likely to be female and African-American or Asian compared to patients with JIA or JDM. Other differences were found between groups related to quality of life measures and ANA positivity.

**Table 1:** Comparison demographics groups among disease groups

Characteristic	Total	JIA	SLE	JDM	*p*-value
Total number	5,756	4,677	639	440	
Female gender N (%)	4,218 (73.3)	3,368 (79.9)	532 (83.4)	318 (72.3)	<0.001
Age at enrollment (years)	11.9 ± 4.8	11.4 ± 4.8	16.0 ± 3.2	11.0 ± 4.4	<0.001
Age onset of symptoms (years)	7.1 ± 4.7	6.5 ± 4.5	12.3 ± 3.2	6.6 ± 3.8	<0.001
Race N (%)					
*AA* ^a^	551 (9.6)	269 (5.8)	225 (35.2)	57 (13.0)	<0.001
*AI* ^b^	95 (1.7)	73 (1.6)	12 (1.9)	10 (2.3)	0.476
*Asian*	197 (3.4)	116 (2.5)	69 (10.8)	12 (2.7)	<0.001
*NH* ^c^	36 (0.6)	25 (0.5)	8 (1.3)	3 (0.7)	0.096
*White*	4,912 (85.3)	4,244 (90.7)	307 (48.0)	361 (82.1)	<0.001
*Other*	222 (3.9)	146 (3.1)	51 (8.0)	25 (5.7)	<0.001
Ethnicity (Non-Hispanic) N (%)	5,059 (87.9)	4,214 (90.1)	464 (72.6)	381 (86.6)	<0.001
Overall Well-being Score	2.3 ± 2.3	2.3 ± 2.3	2.6 ± 2.5	2.1 ± 2.3	<0.001
PGA	1.6 ± 1.9	1.6 ± 1.9	2.0 ± 1.9	1.6 ± 1.9	<0.001
CHAQ	0.34 ± 0.51	0.35 ± 0.51	0.24 ± 0.46	0.36 ± 0.63	<0.001
ANA N (% positive)^d^	2,802 (56.2)	2,012 (49.7)	577 (96.5)	213 (62.3)	<0.001

^a^AA – African American or Black, ^b^AI- American Indian or Alaskan Native

^c^NH- Native Hawaiian or Pacific Islander, ^d^Indicates missing data

#### Family history of autoimmune disease

**Fig. 1:**
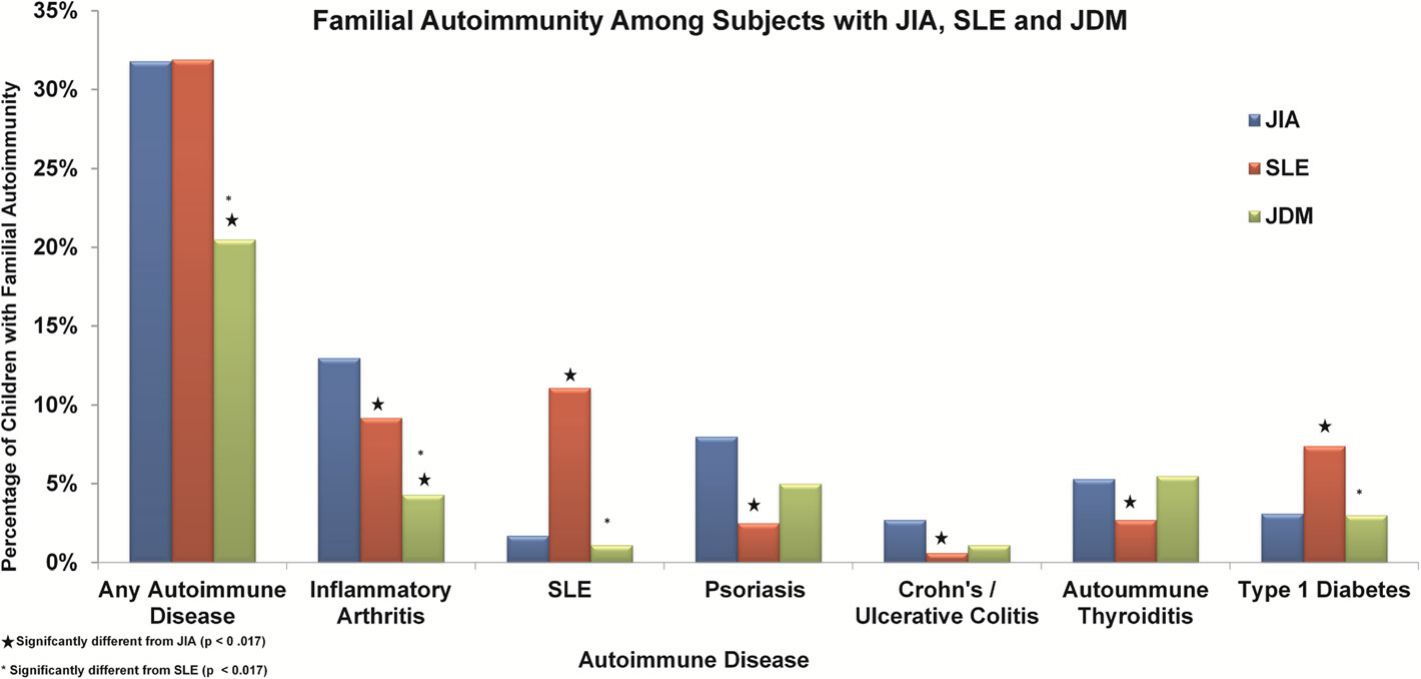
Familial autoimmunity among subjects with JIA, cSLE and JDM. Proportion of cases with JIA, cSLE and JDM that report having first degree relatives with various autoimmune disorders. Asterisks denote differences that are statistically significant after correction for multiple testing

We compared the proportion of children with first-degree relatives with various autoimmune diseases among the three diagnosis groups (Fig. 1 and Table 2). The proportion of patients having first degree relatives with any autoimmune disease in the JDM group (20.5 %) was significantly less compared to patients with JIA (31.8 %, *p* < 0.001) and patients with cSLE (31.9 %; *p* < 0.001). There were no statistically significant differences in the proportion of JIA, cSLE and JDM patients with first degree relatives that had autoimmune thyroiditis, multiple sclerosis, acute anterior uveitis or other autoimmune disorders.

**Table 2:** Comparison of cases with JIA, cSLE and JDM with autoimmune disorders among first-degree relatives

Family history of disease	Disease group			*P*-value
	JIA	cSLE	JDM	
	(*N* = 4,677)	(*N* = 639)	(*N* = 440)	
Any autoimmune disease	1,488 (31.8)	204 (31.9)	90 (20.5)^a^ ^b^	<0.001
Inflammatory Arthritis -(AS, Spondyloarthropathy, JIA, RA)	608 (13.0)	59 (9.2)^a^	19 (4.3)^a^ ^b^	<0.001
Ankylosing spondylitis	61 (1.3)	1 (0.2)^a^	2 (0.5)	0.014
Spondyloarthropathy	36 (0.8)	0 (0.0)	0 (0.0)	0.015
JIA	241 (5.2)	14 (2.2)^a^	4 (0.9)^a^	<0.001
RA	340 (7.3)	50 (7.8)	15 (3.4)^a^ ^b^	0.007
Crohn’s disease or ulcerative colitis	126 (2.7)	4 (0.6)^a^	5 (1.1)	0.002
Acute anterior uveitis	15 (0.3)	0 (0.0)	0 (0.0)	0.169
Psoriasis	372 (8.0)	16 (2.5)^a^	22 (5.0)	<0.001
SLE	77 (1.7)	71 (11.1)^a^	5 (1.1)^b^	<0.001
Multiple Sclerosis	45 (1.0)	7 (1.1)	5 (1.1)	0.905
Autoimmune thyroiditis	248 (5.3)	17 (2.7)^a^	24 (5.5)	0.015
Celiac	36 (0.8)	5 (0.8)	6 (1.4)	0.439
Type 1 Diabetes	147 (3.1)	47 (7.4)^a^	13 (3.0)^b^	<0.001
Other autoimmune	214 (4.6)	32 (5.0)	20 (4.6)	0.893

All values are N (%)

^a^Significantly different from JIA (*p* < 0.017)

^b^Significantly different from SLE (*p* < 0.017)

#### JIA versus cSLE

Compared to subjects with cSLE, a significantly greater proportion of patients with JIA had first-degree relatives with any inflammatory arthritis (*p* = 0.007), ankylosing spondylitis (*p* = 0.016), JIA (*p* = 0.001), psoriasis (*p* < 0.001), Crohn’s disease or ulcerative colitis (*p* = 0.002), and autoimmune thyroiditis (*p* = 0.005). In contrast, compared to JIA, a significantly higher proportion of patients with cSLE had first-degree relatives with SLE (*p* < 0.001) and type-I diabetes (*p* < 0.001). A similar proportion of cases with JIA and cSLE had FDR with RA (7.3 % vs 7.8 %, *p* = ns).

#### JIA versus JDM

Compared to patients with JDM, a significantly greater proportion of patients with JIA had first-degree relatives with JIA (*p* < 0.001), RA (*p* = 0.002), and any inflammatory arthritis (*p* < 0.001) (Table 2).

#### cSLE versus JDM

Compared to patients with JDM, a significantly higher proportion of patients with cSLE had first-degree relatives with any inflammatory arthritis (*p* = 0.003), RA (*p* = 0.003), SLE (*p* < 0.001), and type-1 diabetes (*p* < 0.001) (Fig. 1 and Table 2).

We also calculated the mean and median number of familial autoimmune diseases a child had for each diagnosis group (Table 3). Overall, there was a difference in the distribution of the number of familial autoimmune diseases with JDM having significantly fewer familial diseases compared to JIA (*p* < 0.001) and cSLE (*p* < 0.001). In addition, after classifying the number of familial diseases into 0, 1–2, and 3 or more, we showed that children with JIA or cSLE were more likely to have at least 1 familial autoimmune disease reported compared to children with JDM (*p* < 0.001).

**Table 3:** Comparison of the number of autoimmune disease (family history) among disease groups

Outcome	Level	Disease group			*P*-value
		JIA	SLE	JDM	
		(*N* = 4,677)	(*N* = 639)	(*N* = 440)	
# of autoimmune diseases in family					
* Mean ± SD*	--	0.4 ± 0.8	0.4 ± 0.7	0.3 ± 0.6	<0.001
* Median*		0	0	0	
* (Range)*		(0 – 14)	(0 – 5)	(0 – 4)	
# of autoimmune diseases in family	0	3189 (66.2 %)	435 (68.1 %)	350 (80.8 %)	<0.001
	1-2	1377 (29.4 %)	192 (30.1 %)	82 (18.6 %)	
	3+	111 (2.4 %)	12 (1.9 %)	8 (1.8 %)	

### Family history of autoimmunity among subjects with JIA categories

We then compared the proportion of familial autoimmunity among first-degree relatives of children with the different JIA categories (Table 4). Overall, there were significant differences in the proportion of any familial autoimmunity among the JIA categories (*p* < 0.001). Additionally, among the categories, there was a significant difference in the proportion of children with first-degree relatives with a history of psoriasis (*p* < 0.001), ankylosing spondylitis (*p* < 0.001), spondyloarthropathy (*p* < 0.001), Crohn’s disease or ulcerative colitis (*p* = 0.001), acute anterior uveitis (*p* = 0.005), JIA (*p* = 0.047), RA (*p* < 0.001), and any inflammatory arthritis (*p* < 0.001).

**Table 4:** Comparison of cases with different JIA categories with autoimmune disorders among first-degree relatives

Family history of disease	JIA category^a^								*P*-value^b^
	(*N* = 4441)								
	Systemic	Poly RF(−)	Poly RF(+)	Oligo persistent	Oligo extended	Psoriatic	ERA	Undiff	
	(*N* = 355)	(*N* = 1313)	(*N* = 294)	(*N* = 1293)	(*N* = 340)	(*N* = 273)	(*N* = 451)	(*N* = 122)	
Any autoimmune disease	77 (21.7)	408 (31.1)	87 (29.6)	344 (26.6)	97 (28.5)	146 (53.5)	186 (41.2)	54 (44.3)	<0.001
Inflammatory arthritis^c^	26 (7.3)	169 (12.9)	36 (12.4)	137 (10.6)	36 (10.6)	5 2 (19.1)	97 (21.5)	24 (19.7)	<0.001
Ankylosing spondylitis	0 (0.0)	4 (0.3)	1 (0.3)	7 (0.5)	0 (0.0)	2 (0.7)	37 (8.2)	5 (4.1)	<0.001
Spondyloarthropathy	1 (0.3)	2 (0.2)	1 (0.3)	3 (0.2)	0 (0.0)	5 (1.8)	17 (3.8)	2 (1.6)	<0.001
JIA	10 (2.8)	83 (6.3)	10 (3.4)	67 (5.2)	13 (3.8)	13 (4.8)	32 (7.1)	5 (4.1)	0.047
RA	17 (4.8)	95 (7.2)	25 (8.5)	76 (5.9)	24 (7.1)	37 (13.6)	32 (7.1)	16 (13.1)	<0.001
Crohn’s disease or ulcerative colitis	4 (1.1)	35 (2.7)	7 (2.4)	27 (2.1)	6 (1.8)	6 (2.2)	26 (5.8)	6 (4.9)	0.001
Acute anterior uveitis	0 (0.0)	7 (0.5)	0 (0.0)	1 (0.1)	1 (0.3)	0 (0.0)	6 (1.3)	0 (0.0)	0.005
Psoriasis	20 (5.6)	78 (5.9)	11 (3.7)	70 (5.4)	17 (5.0)	96 (35.2)	31 (6.9)	20 (16.4)	<0.001
SLE	7 (2.0)	26 (2.0)	5 (1.7)	17 (1.3)	5 (1.5)	2 (0.7)	7 (1.6)	5 (4.1)	0.338
Multiple Sclerosis	3 (0.9)	14 (1.1)	2 (0.7)	10 (0.8)	6 (1.8)	3 (1.1)	2 (0.4)	3 (2.5)	0.410
Autoimmune thyroiditis	16 (4.5)	74 (5.6)	17 (5.8)	62 (4.8)	22 (6.5)	23 (8.4)	17 (3.8)	6 (4.9)	0.198
Celiac	1 (0.3)	7 (0.5)	0 (0.0)	15 (1.2)	2 (0.6)	0 (0.0)	6 (1.3)	1 (0.8)	0.113
Type 1 Diabetes	12 (3.4)	41 (3.1)	14 (4.8)	32 (2.5)	8 (2.4)	11 (4.0)	14 (3.1)	6 (4.9)	0.406
Other autoimmune	12 (3.3)	68 (5.1)	10 (3.3)	50 (3.8)	12 (3.5)	14 (5.1)	25 (5.4)	6 (4.7)	0.504

^a^children with missing JIA category information were excluded. All values are N (%)

^b^Significance values for overall differences between JIA categories

^c^Inflammatory arthritis includes AS, spondyloarthropathy, JIA and RA

## Discussion

Autoimmune disorders are common, estimated to affect 5-10 % of the population. Clinical and genetic studies support the hypothesis that clinically distinct autoimmune disorders share common genetic susceptibility factors [1, 16, 17]. Familial aggregation of SLE, RA and other autoimmune diseases have been reported in probands with SLE [18]. Previously studies of small cohorts have shown familial aggregation of autoimmunity in JIA, JDM and cSLE [9–11]. In a study of 69 subjects with cSLE, 32 % of subjects had one or more first degree relatives with autoimmune diseases which matches findings from our study [10]. However, there have not been systematic investigations of large cohorts of children with JIA, SLE and JDM to determine if there are specific patterns of familial clustering with these phenotypes.

Our results suggest that the prevalence of autoimmunity is increased among first degree relatives of subjects with JIA and cSLE compared to those of JDM. Relatives of subjects with cSLE had an increased prevalence of SLE, and relatives of subjects with JIA were enriched for inflammatory arthropathies. Relatives of children with cSLE also had a higher prevalence of type-1 diabetes compared to relatives of cases with JIA or JDM. These results highlight the complex nature of familial autoimmunity suggesting that while prevalence of clinically distinct autoimmune phenotypes are increased among family members of cases with JIA, cSLE and JDM, there are distinct patterns of familial clustering of diseases among these phenotypes.

Our results suggest that familial autoimmunity is lower in JDM compared to JIA or cSLE. Since the sample sizes of our JDM and cSLE cohorts were of similar magnitude, we believe that this is a true difference and not influenced by the potential number of relatives. One possible reason for this observation could be due to a potentially greater contribution of environmental factors to JDM compared to JIA or cSLE. While genome wide association studies of JIA [19] and SLE [20, 21] have resulted in the discovery of a number of associated loci both within and outside the MHC region, in inflammatory myopathies, only HLA variants are associated at genome wide levels of significance, although the sample sizes for inflammatory myopathies was modest at 1700 cases [22]. It is also possible that susceptibility genes that underlie JDM might be different compared to JIA and/or cSLE. Emerging studies of multiple autoimmune phenotypes using shared controls might allow for a better understanding of shared genetic associations between clinically distinct phenotypes, which in turn might have implications towards understanding their pathophysiology and therapy [23]. Future investigations of subjects in families with multiple autoimmune disorders to characterize shared genomic, transcriptomic and proteomic factors would further enhance our understanding of the complex relationship between different autoimmune phenotypes.

When we compared the different JIA categories, we found that systemic JIA cases had lower proportion of first degree relatives with any autoimmune disease (21.7 %) compared to other JIA categories, which is consistent with the idea that systemic JIA may be phenotypically and genetically distinct from other JIA categories [24]. Since family history of psoriasis and spondyloarthropathy is part of the criteria for classifying patients into psoriatic JIA and ERA respectively, this could have biased the observed higher prevalence of autoimmunity among relatives of psoriatic JIA and ERA cases. Interestingly the prevalence of SLE, autoimmune thyroiditis, MS, celiac disease and type 1 diabetes did not differ significantly between relatives of different JIA categories.

Our study had several strengths. The data for the CARRA Registry was collected from a majority of pediatric rheumatology centers in the USA, which means our results apply to a broad population of childhood rheumatic diseases. In addition to being the first to compare familial autoimmunity among JIA, cSLE and JDM, our study comprised of large cohorts with JIA, cSLE and JDM. Our JIA cohort was also sufficiently large which allowed us to investigate differences between different JIA categories.

Our study has potential limitations as well. The CARRA Registry did not collect family history of idiopathic inflammatory myopathy specifically. This could have resulted in an underrepresentation of inflammatory myopathy in these families. However, two smaller studies of probands with idiopathic inflammatory myopathies did not find an increased prevalence of first degree relatives with inflammatory myopathy [9, 25]. Furthermore, an “other” category was included which might have expected to capture information if subjects with JDM had increased numbers of relatives with inflammatory myopathies. The CARRA Registry also did not collect individual data on which relative had the autoimmune disorder or the number of first-degree relatives to provide a denominator. We do not believe that this limitation affects our conclusion that compared to first degree relatives of JDM cases, there is increased autoimmunity among first degree relatives of patients with JIA and cSLE. Since families of probands with JIA, cSLE and JDM completed the same baseline demographic forms it is unlikely that there was bias in reporting of first degree relatives with autoimmune disorders between JIA, cSLE and JDM. The lack of data to discriminate autoimmunity status of individual relatives limits us from estimating the prevalence of autoimmunity among relatives or potential parent of origin effects, but we do not believe there to be inherent differences in number of relatives among JIA, cSLE or JDM families. Finally, our subjects are from North America, and majority (85 %) were Caucasian, which might limit the generalizability of the study to other regions and ethnicities.

## Conclusion

In conclusion, we have shown JIA and cSLE cases more often have first-degree relatives with autoimmunity compared to JDM cases. The demonstration that a greater proportion of JIA cases have first-degree relatives with inflammatory arthritis, and a greater proportion of cSLE cases have first-degree relatives with SLE suggests that there are distinct patterns of familial autoimmunity. Future studies, including investigation of genomic and proteomic biomarkers among subjects with different autoimmune phenotypes as well as their relatives would substantially improve our understanding of the complex interplay between different autoimmune phenotypes.
